# A comparative simulation study of AR(1) estimators in short time series

**DOI:** 10.1007/s11135-015-0290-1

**Published:** 2015-12-09

**Authors:** Tanja Krone, Casper J. Albers, Marieke E. Timmerman

**Affiliations:** Heymans Institute for Psychological Research, Psychometrics and Statistics, Grote Kruisstraat 2/1, 9712TS Groningen, The Netherlands

**Keywords:** Time series analysis, Autocorrelation, AR(1), Bayesian MCMC, Misspecification

## Abstract

Various estimators of the autoregressive model exist. We compare their performance in estimating the autocorrelation in short time series. In Study 1, under correct model specification, we compare the frequentist *r*_1_ estimator, C-statistic, ordinary least squares estimator (OLS) and maximum likelihood estimator (MLE), and a Bayesian method, considering flat (B_f_) and symmetrized reference (B_sr_) priors. In a completely crossed experimental design we vary lengths of time series (i.e., *T* = 10, 25, 40, 50 and 100) and autocorrelation (from −0.90 to 0.90 with steps of 0.10). The results show a lowest bias for the B_sr_, and a lowest variability for *r*_1_. The power in different conditions is highest for B_sr_ and OLS. For *T* = 10, the absolute performance of all measurements is poor, as expected. In Study 2, we study robustness of the methods through misspecification by generating the data according to an ARMA(1,1) model, but still analysing the data with an AR(1) model. We use the two methods with the lowest bias for this study, i.e., B_sr_ and MLE. The bias gets larger when the non-modelled moving average parameter becomes larger. Both the variability and power show dependency on the non-modelled parameter. The differences between the two estimation methods are negligible for all measurements.

## Introduction

Time series analysis has been valuable for achieving insight into the nature of longitudinal processes. Especially the autoregressive moving average (ARMA) model (Box and Jenkins 1976) has gained enormous popularity in various research areas. The autoregressive part models the serial dependence between consecutive measurements. The moving average part models the serial dependence between consecutive error terms. The ARMA(*p*, *q*) model is given by:

1 $$y_t=\mu +\sum ^p_{i=1}\phi _iy_{t-i}+\sum ^q_{j=1}\theta _j e_{t-j}+e_t,\qquad e_t\sim N(0, \sigma ^2_e),$$

with $y_t$ the score at time $t\;(t=1,2,\ldots ,T)$, *μ* the population mean, $\phi _i$ the autocorrelation for lag $i\;(i=1,2,\ldots ,p)$, $\theta _j$ the moving average parameter at lag $j\; (j=1,2,\ldots ,q)$ and $e_t$ the residual.

One of the simplest versions of the ARMA(*p*, *q*) is the AR(1) model:

2 $$y_t=\mu +\phi (y_{t-1}-\mu )+e_t,\qquad e_t\sim N(0, \sigma ^2_e),$$

where, for simplicity, the subscript 1 is omitted from *ϕ*. Several estimation methods have been proposed to estimate the AR(1) model. These estimation methods include closed form estimation methods, such as the *r*_1_ estimator (Yule 1927; Walker 1931; Box and Jenkins 1976), C-statistic (Young 1941) and Ordinary Least Squares (OLS) estimator, and iterative estimation methods, such as frequentist Maximum Likelihood Estimation (MLE) and Bayesian Markov Chain Monte Carlo (MCMC) estimation. The performance of the closed form estimation methods in terms of efficiency have been examined and compared in some simulation studies (Huitema and McKean 1991; DeCarlo and Tryon 1993; Arnau and Bono 2001; Solanas et al. 2010). Generally, in particular for shorter time series (e.g., length $T \le 50$), the closed form estimation methods have been shown to have biased autocorrelation estimates and/or high variability. Because the closed form and iterative estimation methods have not been mutually compared so far, it is unclear which estimation methods perform better in terms of having a low bias and variability under relevant conditions for empirical practice. Further, little is known about the robustness of the specific estimation methods towards misspecification of the model. This knowledge is important to optimize a time series research design, and to select a low-variability, low-bias, and robust method for estimating an AR(1) model in empirical practice.

In this paper, we discuss two studies to assess the relative performance of several estimators of the AR(1) model. We focus on short time series, with a length *T* between 10 and 100. Even though these lengths are relevant, for example in psychological research, they are not thoroughly studied yet for all estimators we compare. For the autocorrelation we use values between −1 and 1, and hence consider stationary time series. Our first study provides the information needed to make an informed choice between the estimation methods for the AR(1) model. To this end, we selected five popular and/or promising estimation methods. In a simulation study, we compare these methods with regard to bias, standard error, the bias of the standard error, the rejection rate for $\phi =0$, the power for $\phi \ne 0$, and the point and 95 % interval estimates.

Our second study focuses on the issue of robustness. Robustness, as used in this paper, is the resilience to misspecification with regard to the number of parameters. The effects of misspecification of the ARMA(1,1), AR(1) and AR(2) model have been studied for the least squares estimator. For an underspecified model, the parameters become more biased when the unspecified parameters are further from zero (Tanaka and Maekawa 1984). Overspecification of the model gives a larger prediction mean squared error for the estimation of the score at $y_t$ (Kunitomo and Yamamoto 1985). To study the robustness with regard to misspecification, we use the two estimation methods that showed the lowest bias in the first study. In the misspecification study we generate the data using an ARMA(1,1) model, but estimate the parameters as if the data was generated using the same AR(1) = ARMA(1,0) model as used in Study 1.

In the next section, we describe the selection process of the estimation methods used in this paper, followed by a short introduction to the estimators. Then, we present the design, performance criteria and the results of the first simulation study, which aims at comparing various estimators when applied to short time series following an AR(1) model. We continue with the design and results of the second simulation study, which aims at exploring the effect of underspecifying a short time series following an ARMA(1,1) model as data following an AR(1) model. We conclude our paper with a discussion of the simulation studies and the implications of the results.

**Table 1:** List of papers with considered estimators, the lengths of the time series, and the outcome measures, where *ϕ* autocorrelation, *th* theoretical, *emp* empirical, *av* averaged over all *ϕ*, and *av+* averaged over all positive *ϕ*. All papers used a range of simulated autocorrelations of [−0.9 (0.1) 0.9], the estimators with ‘*r*’ in their name are derived from *r*_1_ estimator

Paper	Estimators	Length	Outcome measures
Huitema and McKean (1991)	*r*_1_, $r_1^+$, $r_1*$, $r_1^c$, $r_1^e$, OLS	6, 10, 20, 50, 100, 500	Bas (th & emp), MSE (av+), α, power, $\sigma _e^2$ (th & emp)
DeCarlo and Tryon (1993)	$r_1$, $r_1^+$, C	6, 10, 20, 30, 50	Bias (emp), MSE (av+), α, power
Huitema and McKean (1994)	$r_{q1}$, $r_{q2}$, $r_{q3}$	6, 10,20, 50, 100, 500	Bias (emp, av & *ϕ*=0.9), MSE (av), α, power, $\sigma _e^2$ (emp)
Arnau and Bono (2001)	$r_1$, $r_1^+$, $r_1'$	6, 10, 20, 30, 50	Bias (th & emp), MSE (av), α, power
Solanas et al. (2010)	$r_1$,$r_1^+$, $r_1*$, $r_1^c$, $r_1^e$, C, $r_1^f$, OLS, $r_1^{fb}$,$r_1^{\delta }$	5, 6, 7, 8, 9, 10, 15, 20, 50, 100	Bias (emp), MSE, α, power

## Selection of estimation methods

To start, we performed a literature search towards estimation methods for AR(1) models. Our selection criteria for the papers were as follows: (1) it must discuss one or more simulation studies that compare different estimators of the AR(1) model; (2) it must include conditions with less than 50 time points and a range of values between *r*_1_ and 1 for the autocorrelation *ϕ*. This literature search revealed the five papers shown in Table 1.

The earliest discussed estimator is the *r*_1_ estimator (Walker 1931), as implemented in the Yule–Walker model (Yule 1927; Box and Jenkins 1976). However, since several studies have found that the bias of *r*_1_ for small samples is large, especially for data with a positive autocorrelation, various alternatives were proposed (Huitema and McKean 1991, 1994; DeCarlo and Tryon 1993; Arnau and Bono 2001; Solanas et al. 2010). A selection of these is given by name in Table 1. Note that most alternatives are based on the original *r*_1_, as can be deduced from the names using ‘*r*’ or ‘*r*_1_’ and a sub- or superscript. In general, the modifications of *r*_1_ showed a smaller bias than *r*_1_ itself, but a larger variability of the estimated autocorrelation (Huitema and McKean 1991, 1994; Arnau and Bono 2001; Solanas et al. 2010), except for the estimators $r^+_1$ and the C-statistic. In direct comparisons between $r^+_1$ and the C-statistic, it was shown that the C-statistic had a smaller average bias and a smaller average mean square error, thus a smaller variability, over different values of *ϕ* than the $r^+_1$ estimation method.

Apart from the modifications of *r*_1_, another closed form solution may be used. The ordinary least squares (OLS) estimator is used in many different applications, most notably in regression analysis. Since the autocorrelation may be interpreted as a special kind of regression parameter, OLS can be used to find the autocorrelation. In comparisons, the OLS estimator showed a smaller bias than most derivations from the *r*_1_ estimators (Huitema and McKean 1991; Solanas et al. 2010). However, the OLS estimator also showed a slightly larger mean squared error than most *r*_1_ derivations. These comparisons between estimators reveal a bias-variance tradeoff in the autocorrelation estimator.

Two important methods that are not found in the comparisons listed in Table 1, are the frequentist MLE and Bayesian MCMC estimation. Though simulation studies using MLE have been done, those studies did not include the conditions of our primary interest. For example, the studies that considered different ARMA(*p*, *q*)-models (Stoica et al. 1986; Pantula and Fuller 1985; Garcia-Hiernaux et al. 2009 ), had no condition with less than 100 time points (Cox and Llatas 1991) or were aimed at examining other parts of the estimation process, such as deciding on which ARMA(*p*, *q*)-model to use (Watson and Nicholls 1992). This was the same for papers using Bayesian MCMC estimation. Examples of this are studies that have no systematic comparison using different estimators (Price 2012), use AR(2) models (West and Wilcox 1996) or use lagged cross-correlation (Zhang and Nesselroade 2007). The MLE and Bayesian MCMC have become often-used methods of analysis in different fields and applications.

## Estimation methods

In the next paragraphs we will describe the five different estimation methods used in this paper.

### The *r*_1_ estimator in the Yule–Walker method

The Yule–Walker method for ARMA models (Yule 1927; Walker 1931; Box and Jenkins 1976) may be the best known estimation method in time series analysis. It uses the *r*_1_ estimator to estimate the lag 1 autocorrelation:

$$\hat{\phi }_{r1}=\frac{\sum _{t=1}^{T-1}\left( y_t-\bar{y}\right) \left( y_{t+1}-\bar{y}\right) }{\sum _{t=1}^T\left( y_t-\bar{y}\right) ^2},$$

where $y_t$ is the observed score at time *t*, $(t=1,2,\ldots ,T)$ and $\bar{y}$ is the mean score over the *T* observations. Asymptotically, the autocorrelation function for this series is biased by $-(1+4 \phi )/T$ (Kendall and Ord 1990). This bias has empirically been shown to be as large as −0.73 for *T* = 6 and $\phi = 0.90$ (DeCarlo and Tryon 1993). This empirical bias is surprisingly close to the asymptotic bias of −0.77. To keep the bias within reasonable limits, Box and Jenkins (1976, pp. 32–33) advise a minimum length of 50 time points for a time series.

The standard error of the $\hat{\phi }_{r_1}$ is calculated as:

3 $$SE_{r_1}=\sqrt{\frac{\hat{\sigma }_e^2}{(T-1)\hat{\sigma }^2_ y}},$$

where $\hat{\sigma }^2_y$ is the estimated variance of $y_t$ and $\hat{\sigma }_e^2$ is the estimated variance of *e*.

In comparison studies, several other proposals were done to replace the *r*_1_ estimator (Huitema and McKean 1991, 1994; Young 1941). One of these, which outperformed the *r*_1_ estimator and some of the other estimators in several studies, was the C-statistic (Young 1941; DeCarlo and Tryon 1993; Solanas et al. 2010).

### C-statistic

The C-statistic (Young 1941) compensates the bias of the *r*_1_ estimator by adding a factor to $\hat{\phi }_{r1}$ as:

$$\hat{\phi }_C=\hat{\phi }_{r1}+\frac{\left( y_T-\bar{y}\right) ^2\left( y_{1}-\bar{y}\right) ^2}{2\sum _{t=1}^T\left( y_t-\bar{y}\right) ^2}.$$

The $\hat{\phi }_C$ is asymptotically unbiased. The $\hat{\phi }_{C}$ has been shown to be a better estimator than $\hat{\phi }_{r1}$ for *ϕ* for short time series and a positive *ϕ* (DeCarlo and Tryon 1993; Solanas et al. 2010)). However, the bias still remains quite large (e.g., −0.38 for $\phi =0.60$ and *r* = 5) and the power remains quite low (e.g., $\le 0.09$ for $\phi =0.60$ and *r* = 5) for short time series (Solanas et al. 2010).

The standard error associated with $\hat{\phi }_C$ is:

4 $$SE_C=\sqrt{\frac{T-2}{(T-1)(T+1)}},$$

which is obviously only dependent on the number of observations.

### Ordinary least squares

The ordinary least squares (OLS) for an AR(1) model is:

$$\hat{\phi }_{ols}=\frac{\sum _{t=1}^{T-1}\left( y_t-\bar{y}\right) \left( y_{t+1}-\bar{y}\right) }{\sum _{t=1}^{T-1}\left( y_t-\bar{y}\right) ^2}.$$

The asymptotic standard error for $\hat{\phi }_{ols}$ is:

5 $$SE_{ols}=\sqrt{\frac{T-(T-1)\phi ^2-1}{T^2-T-Ty_T^2}}.$$

The OLS estimation is capable of handling non-stationary data under certain restrictions. This means that it is possible to obtain a non-stationary estimate (i.e., $|\hat{\phi }_{ols}|> 1$). To identify possible different behaviours, we distinguish two types of OLS analysis results: OLS-A will refer to the complete results, where OLS-S will refer to the results where the non-stationary results are left out.

### Maximum likelihood estimation

The iterative Maximum Likelihood Estimation (MLE) used to estimate the autocorrelation, shares asymptotic properties with the OLS estimation (Lütkepohl 1991, p. 368–370). The MLE method uses a collection of algorithms to find the maximum likelihood for a parameter or model (Durbin and Koopman 2012). In this study, we will compute the MLE with the ‘Broyden–Fletcher–Goldfarb–Shanno’ algorithm (Byrd et al. 1995). An asymptotic standard error for $\hat{\phi }_{mle}$ may be estimated in the same way as for $\hat{\phi }_{r_1}$, using Eq. 3. The asymptotic bias for an AR(1) model with population mean assumed to be zero, is $-2\phi /T$. For an AR(1) model with the mean estimated, the asymptotic bias is $(-3\phi +1)/T$ (Tanaka 1984).

### Bayesian Markov Chain Monte Carlo

The Bayesian MCMC is the only non-frequentist estimation method considered in this paper. Bayesian analysis uses a prior probability distribution for the parameters, set up before the analysis. This is combined with the observed likelihood, as computed from the observed data, to form the posterior probability of the parameters. This posterior probability can be expressed through Bayes’ theorem: $p(\phi |Y)\propto (Y|\phi )p(\phi )$. For the Bayesian analyses we will use MCMC sampling to find the combination of parameter values which gives the highest likelihood.

In these simulation studies we will consider two weak informative Bayesian priors. Since we assume stationarity we restrict ourselves to prior distributions with non-zero probabilities for $|\phi |\le 1$. That is, we consider a flat prior, giving all values of *ϕ* between −1 and 1 an equal probability:

$$\begin{aligned}\pi _{f(\phi )} = \textstyle {\frac{1}{2}}, \quad&\text {for } -1 \le \phi \le 1. \end{aligned}$$

Further, we consider the symmetrized reference prior defined by (Berger and Yang 1994), which is specifically tailored to autoregressive processes. The symmetrized reference prior is given as:

$$\begin{aligned} \pi _{sr(\phi )} = 1/[2\pi \sqrt{1-\phi ^2}], \quad&\text {for } -1 \le \phi \le 1. \end{aligned}$$

This symmetrized reference prior gives a higher probability to higher values of $|\phi |$ and has a narrower posterior distribution and a smaller mean square error than the flat prior or Jeffrey’s prior in the case of AR(1) models (Berger and Yang 1994). We will denote these methods as B_f_ and B_sr_, respectively.

## Research design study 1: comparison of estimators

To compare the various estimators for the autocorrelation (*ϕ*), we simulate according to an AR(1) model (see Eq. 2). In the generation of the data we vary the length of the time series *T* and the autocorrelation *ϕ*. For *T* we use five different sizes, namely 10, 25, 40, 50 and 100. For $\phi$, we use an autocorrelation of −0.90 to 0.90 inclusive, taking steps of 0.10. Earlier studies show that there is a difference between the bias for the negative and positive *ϕ* for several estimators, including *r*_1_ and the C-statistic (DeCarlo and Tryon 1993; Solanas et al. 2010). This indicates that a thorough test is required to include both positive and negative autocorrelations. Finally, the number of replications must be set. All of the studies in Table 1 have a minimum of 10,000 replications per condition. However, a pilot study showed that the maximum standard deviation of the mean $\hat{\phi }$ over 5000–10,000 replications was 0.0007, when *T* = 10 and $\phi =0.7$, for all estimators. Therefore we use *N* = 2000 replications per condition. Considering a fully crossed experimental design, this yields 19 × 5 × 5000 = 475,000 simulated data sets.

Across all conditions, *μ* is set to zero and $\sigma ^2_e$ to one, which can be done without loss of generality. This results in a standard normal distribution for $y_t$ given *ϕ*.

**Table 2:** Different combinations of priors tested to see their influence on the posterior results, with the used prior distributions (top) and parameters as estimated (with the empirical standard deviation) with these distributions (bottom)

Parameter	Test 1		Test 2		Test 3		Test 4		Test 5		Test 6		Test 7	
Priors used														
*μ*	N(0,2)		N(1,2)		N(0,5)		N(1,5)		N(0,2)		N(0,2)		N(0,2)	
$\sigma _e$	$\Gamma (2,2)$		$\Gamma (2,2)$		$\Gamma (2,2)$		$\Gamma (2,2)$		$\Gamma (1,1)$		$\Gamma (1,2)$		$\Gamma (2,1)$	
Mean estimated parameters and their standard deviation in brackets for $\phi =-0.50$														
B_f_: *ϕ*	−0.33 (0.29)		−0.32 (0.29)		−0.31 (0.30)		−0.31 (0.30)		−0.32 (0.29)		−0.34 (0.29)		−0.30 (0.29)	
B_f_: *μ*	0.00 (0.22)		0.05 (0.23)		0.01 (0.25)		0.03 (0.25)		0.00 (0.22)		0.00 (0.22)		0.01 (0.22)	
B_f_: $\sigma _e$	1.06 (0.24)		1.06 (0.24)		1.06 (0.24)		1.06 (0.24)		1.07 (0.26)		0.99 (0.23)		1.16 (0.28)	
B_sr_: *ϕ*	−0.37 (0.34)		−0.37 (0.34)		−0.34 (0.37)		−0.34 (0.37)		−0.37 (0.34)		−0.39 (0.34)		−0.34 (0.34)	
B_sr_: *μ*	0.01 (0.22)		0.07 (0.24)		0.01 (0.27)		0.06 (0.28)		0.00 (0.22)		0.00 (0.22)		0.01 (0.22)	
B_sr_: $\sigma _e$	1.06 (0.24)		1.06 (0.24)		1.07 (0.24)		1.07 (0.24)		1.08 (0.26)		1.00 (0.23)		1.16 (0.28)	
Mean estimated parameters and their standard deviation in brackets for $\phi =0$														
B_f_: *ϕ*	0.05 (0.29)		0.05 (0.30)		0.08 (0.31)		0.08 (0.31)		0.05 (0.29)		0.04 (0.30)		0.07 (0.28)	
B_f_: *μ*	0.00 (0.32)		0.12 (0.33)		−0.00 (0.40)		0.06 (0.40)		0.00 (0.32)		0.00 (0.33)		0.00 (0.32)	
B_f_: $\sigma _e$	1.05 (0.23)		1.06 (0.23)		1.07 (0.23)		1.07 (0.23)		1.07 (0.25)		0.99 (0.22)		1.15 (0.26)	
B_sr_: *ϕ*	0.08 (0.36)		0.09 (0.36)		0.14 (0.38)		0.14 (0.38)		0.08 (0.36)		0.06 (0.36)		0.10 (0.35)	
B$_{\text {sr}}$: $\mu$	0.00 (0.31)		0.18 (0.33)		0.00 (0.43)		0.13 (0.44)		0.00 (0.31)		0.00 (0.32)		0.00 (0.30)	
B$_{\text {sr}}$: $\sigma _e$	1.07 (0.23)		1.07 (0.23)		1.09 (0.24)		1.09 (0.24)		1.09 (0.25)		1.01 (0.22)		1.17 (0.27)	
Mean estimated parameters and their standard deviation in brackets for $\phi =0.50$														
B$_{\text {f}}$: $\phi$	0.38 (0.25)		0.39 (0.25)		0.42 (0.26)		0.42 (0.26)		0.38 (0.25)		0.37 (0.26)		0.38 (0.24)	
B_f_: *μ*	−0.00 (0.56)		0.23 (0.56)		−0.01 (0.74)		0.13 (0.74)		0.00 (0.55)		0.00 (0.57)		0.00 (0.54)	
B_f_: $\sigma _e$	1.02 (0.22)		1.02 (0.22)		1.03 (0.23)		1.03 (0.23)		1.03 (0.24)		0.96 (0.22)		1.11 (0.25)	
B_f_: $\phi$	0.46 (0.28)		0.47 (0.28)		0.53 (0.28)		0.53 (0.28)		0.46 (0.28 )		0.45 (0.29)		0.47 (0.27)	
B_f_: $\mu$	−0.00 (0.51)		0.34 (0.51)		−0.01 (0.75)		0.26 (0.76)		−0.00 (0.51)		−0.00 (0.53)		−0.00 (0.49)	
B_f_: $\sigma _e$	1.03 (0.22)		1.04 (0.22)		1.05 (0.23)		1.05 (0.23)		1.05 (0.24)		0.97 (0.22)		1.12 (0.25)	

*Priors* We performed a small simulation study to decide on the values for the hyperparameters of the priors in our Bayesian analyses. In the model we use, only the prior distributions for *μ* and $\sigma _e$ have such hyperparameters. We used 3 conditions, with $\phi =-0.50,\;0$ and 0.50, using 1000 replications per condition and 2000 iterations per analysis. We set *T* = 10, since shorter series provide less data, and will therefore be more strongly influenced by the choice of the prior. For *μ* we used a normal prior with mean and standard deviation as given, and for $\sigma _e$ we used a γ prior with shape and rate as given in the top part of Table 2.

As can be seen in Table 2, the differences in the estimated parameters are small, especially when taking into account the uncertainty added by the small *T*. As a result, we based our choice of priors on theoretical grounds. To reduce the influence of the priors, we choose our priors close to the distributions used for the data generation: $\mu \sim N(0,2)$ and $\sigma _e \sim \Gamma (2,2).$

*Outcome measures* For each data set we obtain different estimators: *r*_1_, C-statistic, OLS, MLE, B_f_ and B_sr_. To compare the estimators, we consider the bias of the various estimators of *ϕ*, their empirical standard error, the bias of the estimated standard error, the rejection rate for $\phi =0$, power for $\phi \ne 0$, and the point and 95 % interval estimates of *ϕ*. All outcome measures are calculated for each condition and each estimation method.

### Bias

The bias is computed as:

$${\text {Bias}} = \left( \frac{1}{N}\sum _{n=1}^{N}\hat{\phi }_{n}\right) - \phi ,$$

where $n \; (n=1,2,\ldots ,N)$ refers to the replication number.

### Variability

To compare the variability of the different estimators over the different conditions, we consider two estimators: the empirical standard error and the bias of the estimated standard error. The empirical standard error shows the variability of the $\hat{\phi }$ across replications. The bias of the estimated standard error shows to what extent the standard error estimated by the estimation method, resembles the empirical standard error.

#### Empirical standard error: $SD(\hat{\phi })$

The empirical standard error of $\hat{\phi }$ is calculated by:

$$SD(\hat{\phi }) = \sqrt{\frac{1}{N-1}\sum _{n=1}^{N} \left( \hat{\phi }-\bar{\hat{\phi }}\right) ^2},$$

where $\bar{\hat{\phi }}$ is the mean estimated $\hat{\phi }$ over all replications within a condition.

#### Bias of the estimated standard error

For the frequentist estimators, the estimated standard error $SE(\hat{\phi })$ is calculated using Eqs. 3, 4 and 5, and for the Bayesian estimation, the estimated standard error is obtained through MCMC. To estimate the expected value of $\overline{SE}(\hat{\phi })$ for each estimator, we compute the average $SE(\hat{\phi })$ over all replications within a condition:

$$\overline{SE}(\hat{\phi }) = \frac{1}{N}\sum _{n=1}^N SE(\hat{\phi }).$$

To assess the bias of the estimated standard error with regard to the observed standard error, we substract the observed standard error, $SD(\hat{\phi })$ from the mean estimated standard error, $\overline{SE}(\hat{\phi })$:

$${\text {Bias}}\;{\text{of }} \; SE(\hat{\phi }) = \overline{SE}(\hat{\phi }) - SD(\hat{\phi }).$$

### Rejection rate and power

For each estimation method and condition, we compute the empirical probability (EPr) for rejecting $H_0:\phi =0$, with $\alpha =0.05$. In the condition with $\phi =0$, the EPr indicates the rejection rate or actual α, in all other conditions the EPr equals the actual power. For the *r*_1_, MLE, OLS-S and C-statistic methods, first a *p* value is obtained using a *t*-distribution. Considering the *t*-statistic for a correlation coefficient:

$$t_{\text {all}} = \frac{\hat{\phi }\sqrt{T-2}}{\sqrt{1-\hat{\phi }^2}},\quad df_{\text {all}}= T-3.$$

For the OLS-A method, a *t* test based on the estimated standard error of $\hat{\phi }$ is applied, since the possibility of $\hat{\phi }$ having a higher value than one in absolute value renders the *t*-statistic for correlations inapplicable:

$$t_{\text {ols}} = \frac{\hat{\phi }}{SE_{\hat{\phi }}}, \quad df_{\text {ols}}= T-3.$$

For the Bayesian estimation methods, we consider the percentage of datasets for which the 95 % credible interval (CrI) does not hold zero.

For each condition and method, we then calculate the EPr of rejecting $H_0: \phi =0$ as:

$$\begin{array}{*{20}l}&{\text {for }}r_1,{\text {C-statistic, MLE, OLS-}} {\text {A, OLS-S:}}& \text{EPr}=\#(H_0 {\text { is rejected}})/N,\\ &{\text {for }}  B_{f}, B_{sr}{:}& {\text{EPr}}=\#({\text {CrI}\, {\text{does not hold}}\,0})/N. \end{array}$$

### Point and interval estimates for *ϕ*

To illustrate the joint effects of bias and variability we consider the two estimation methods with the smallest bias, using the point and interval estimates of $\phi$. As point estimate we use the mean of $\hat{\phi }$ per condition, for the interval estimation we use the mean 95 percentile of the $\hat{\phi }$ over all replications per condition.

### Procedure

For the simulations and analyses we use the program ‘R’ (R Core Team 2014). The C-statistic was computed directly with the basic functions available. For the Yule–Walker, OLS and MLE methods we use the command ‘ar’ from the software package ‘stats’. The Bayesian analyses are done with the program ‘Rstan’ (Stan Development Team 2014).

## Results study 1

The OLS estimator rendered estimates of *ϕ* that were higher than one in absolute value, and thus non-stationary, as expected. The highest percentage of non-stationary estimates, 15.1 %, was found for the shortest series, *r* = 10 and the highest autocorrelation, $\phi =0.90$. For *r* = 10 and $\phi =-0.90 \; \text { to } \; \phi =0.80$, up to 6.8 % of the estimates per condition were non-stationary, with higher percentages associated with higher values of $|\phi |$. For *T* = 25 to 50 and $\phi = 0.50$ to 0.90 in absolute value, up to 2.3 % of the estimates were non-stationary. However, the difference in the results was quite small. Thus we will discuss only the OLS-A results for the OLS, which includes all measurements, unless the OLS-S shows a strong deviation from OLS-A.

For the Bayesian analysis, non-convergence is expressed in the potential scale reduction factor, $\hat{R}$. The potential scale reduction factor shows the ratio of how much the estimation may change when the number of iterations is doubled, with a perfect 1 indicating that no change is expected (Gelman and Rubin 1992; Stan Development Team 2014). For each estimated parameter $\phi$, *μ* and $\sigma _e$, less than 0.39 % of the estimates showed a $\hat{R}$ above 1.02. Furthermore, a maximum of $2.8\,\%$, found for *μ* as estimated with B_f_, showed a $\hat{R}$ above 1.01.

### Bias

**Fig. 1:**
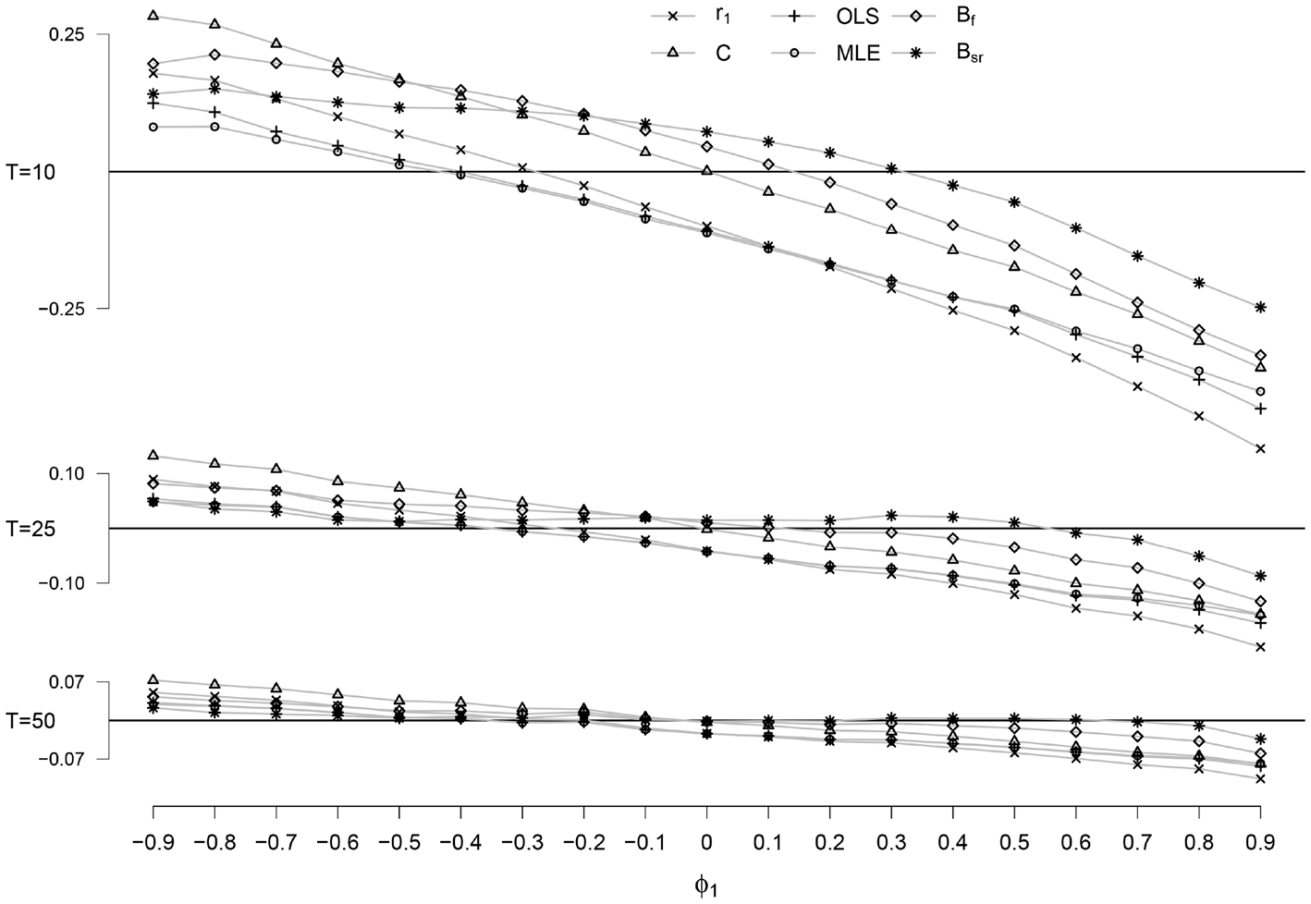
Bias for the six estimators and time series lengths *T* = 10, 25 and 50 as a function of *ϕ*

The bias of the six estimators as a function of *ϕ* for *T* = 10, 25, and 50 is presented in Fig. 1. The conditions for *T* = 40 and are not shown due to their uninformative nature: *T* = 40 yields results highly similar to *T* = 50, and *T* = 100 yields results with hardly any differences between the estimators. As can be seen in Fig. 1, the bias becomes smaller as *T* increases for all methods, which is to be expected. The relation between the bias and *ϕ* is roughly linear for all methods, being positive for negative values of *ϕ* and negative for positive values of *ϕ*. Further, the bias for positive values of *ϕ* is larger than the bias for their negative counterparts (i.e. −*ϕ*). This holds for all values of *T* and for all methods, except for the C-statistic.

With regard to the ordering of the estimation methods, differences are found between negative and positive values of *ϕ* and between short time series, *T* = 10, and longer time series, *T* ≥ 25. For the shortest time series with *T* = 10, the differences between the methods with regard to bias are strongly dependent on *ϕ*. For low, negative values of *ϕ*, the smallest bias is shown by the OLS, MLE and, to a lesser extent, the *r*_1_. For positive values of *ϕ*, the smallest bias is shown by the B_sr_, followed by the B_f_. The largest bias for *T* = 10 is associated with the C-statistic for negative values of *ϕ*, and the *r*_1_ for positive values of *ϕ*.

For *T* ≥ 25 and any *ϕ*, B_sr_ consistently shows the smallest bias. Just as for the shortest series, the largest bias for *T* ≥ 25 is associated with the C-statistic for negative values of *ϕ*, and with the *r*_1_ for positive values of *ϕ*.

**Fig. 2:**
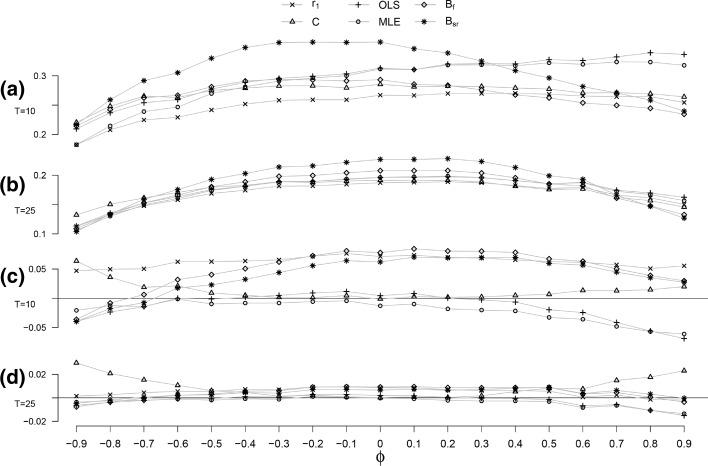
The empirical standard error for *T* = 10 (*panel a*) and *T* = 25 (*panel b*), and the bias of the estimated standard error for *T* = 10 (*panel c*) and *T* = 25 (*panel d*), as a function of *ϕ* by estimation method

### Variability

With regard to variability, the results for $T\ge 40$ are highly similar to the results for *T* = 25 with regard to pattern of the variability and the order of the estimation methods. The only difference is the decline in absolute size. This prompted us to only explicitly show the results for *T* = 10 and *T* = 25 for the empirical standard error and the bias of the estimated standard error.

#### Empirical standard error: $SD(\hat{\phi })$

The empirical standard error ($SD(\hat{\phi })$) as a function of *ϕ* is shown in Fig. 2 for *T* = 10 (panel a) and *T* = 25 (panel b). For all frequentist estimators, the $SD(\hat{\phi })$ for positive values of *ϕ* is larger than the $SD(\hat{\phi })$ for their negative counterparts (i.e. −*ϕ*), implying that the variability is higher for positive values of *ϕ* than for negative values of *ϕ*. For the Bayesian estimators, this differs between values of *T* and $|\phi |$.

With regard to the ordering of the estimation methods for the $SD(\hat{\phi })$, small differences are found between the short time series, *T* = 10, and longer time series, $T \ge 25$. For the shortest time series, *T* = 10, and *ϕ* below 0.40, the lowest $SD(\hat{\phi })$ is shown by *r*_1_, for *ϕ* above 0.40 this is shown by B_f_. The highest $SD(\hat{\phi })$ for *ϕ* below 0.30 is shown by B_sr_, for *ϕ* above 0.30 this is shown by the OLS estimator. The OLS and MLE stand out due to the continuing increase in the $SD(\hat{\phi })$ for higher values of *ϕ*.

For *T* ≥ 25, the B_sr_ shows an distinct pattern. The B_sr_ shows the lowest $SD(\hat{\phi })$ for *ϕ* below −0.80 and above 0.80, but the highest $SD(\hat{\phi })$ for *ϕ* between −0.6 and 0.60. The lowest $SD(\hat{\phi })$ for *ϕ* between —0.70 and 0.40 is shown by the *r*_1_. The highest $SD(\hat{\phi })$ for *ϕ* below −0.70 is shown by the C-statistic, for *ϕ* above 0.70 this is shown by the OLS followed by the MLE. When *T* increases, the empirical standard error of the different methods become smaller and more similar to each other. For *T* = 100, the size of the $SD(\hat{\phi })$ is between 0.05 and 0.10 for all values of *ϕ* and all estimators.

#### Bias of the estimated standard error

The bias of $SE(\hat{\phi })$ as a function of *ϕ* is shown in Fig. 2 for *T* = 10 (panel c) and for *T* = 25 (panel d). In general, the bias of $SE(\hat{\phi })$ decreases when *T* becomes larger, indicating a smaller difference between the estimated and the empirical standard errors. For *T* = 100, the bias of $SE(\hat{\phi })$ is between −0.01 and 0.04 for all values of *ϕ* and all estimators. With regard to the ordering of the estimation methods, small differences are found between *T* = 10 and longer time series. Differences were also found for different values of *ϕ*.

The direction of the bias of $SE(\hat{\phi })$ differs between the methods and the value of *ϕ*. For *r*_1_ and the C-statistic, the bias of $SE(\hat{\phi })$ is positive for all *ϕ*, indicating an overestimation of the standard error. The OLS shows a positive bias of $SE(\hat{\phi })$ for *ϕ* between −0.70 and 0.20, and a negative bias of $SE(\hat{\phi })$ for other values of *ϕ*. For the MLE the bias of $SE(\hat{\phi })$ is negative for all *ϕ*. Both B_f_ and B_sr_ show a negative bias of $SE(\hat{\phi })$ for $\phi < -0.70$, and a positive bias of $SE(\hat{\phi })$ for higher values of *ϕ*.

For T = 10, the smallest bias of $SE(\hat{\phi })$ for *ϕ* below −0.70 is shown by the OLS method. The smallest bias of $SE(\hat{\phi })$ for *ϕ* above −0.50 is shown by the C-statistic, closely followed by the OLS and the MLE. The largest bias of $SE(\hat{\phi })$ for *ϕ* above −0.80, is shown by *r*_1_, which is joined in this regard by B_f_ and B_sr_ for *ϕ* between −0.20 to 0.60.

The bias of $SE(\hat{\phi })$ for *T* ≥ 25 is smaller than the bias of $SE(\hat{\phi })$ for *T* = 10 and the different methods are closer together. The domain of *ϕ* for which the C-statistic shows the largest bias of all estimators increases when *T* becomes larger; for *T* = 10 this is when *ϕ* is below −0.50 and above 0.70, for *T* = 100 this is when *ϕ* is below −0.30 and above 0.20. The other estimators show the same pattern and order in the bias of $SE(\hat{\phi })$ as for *T* = 10.

**Fig. 3:**
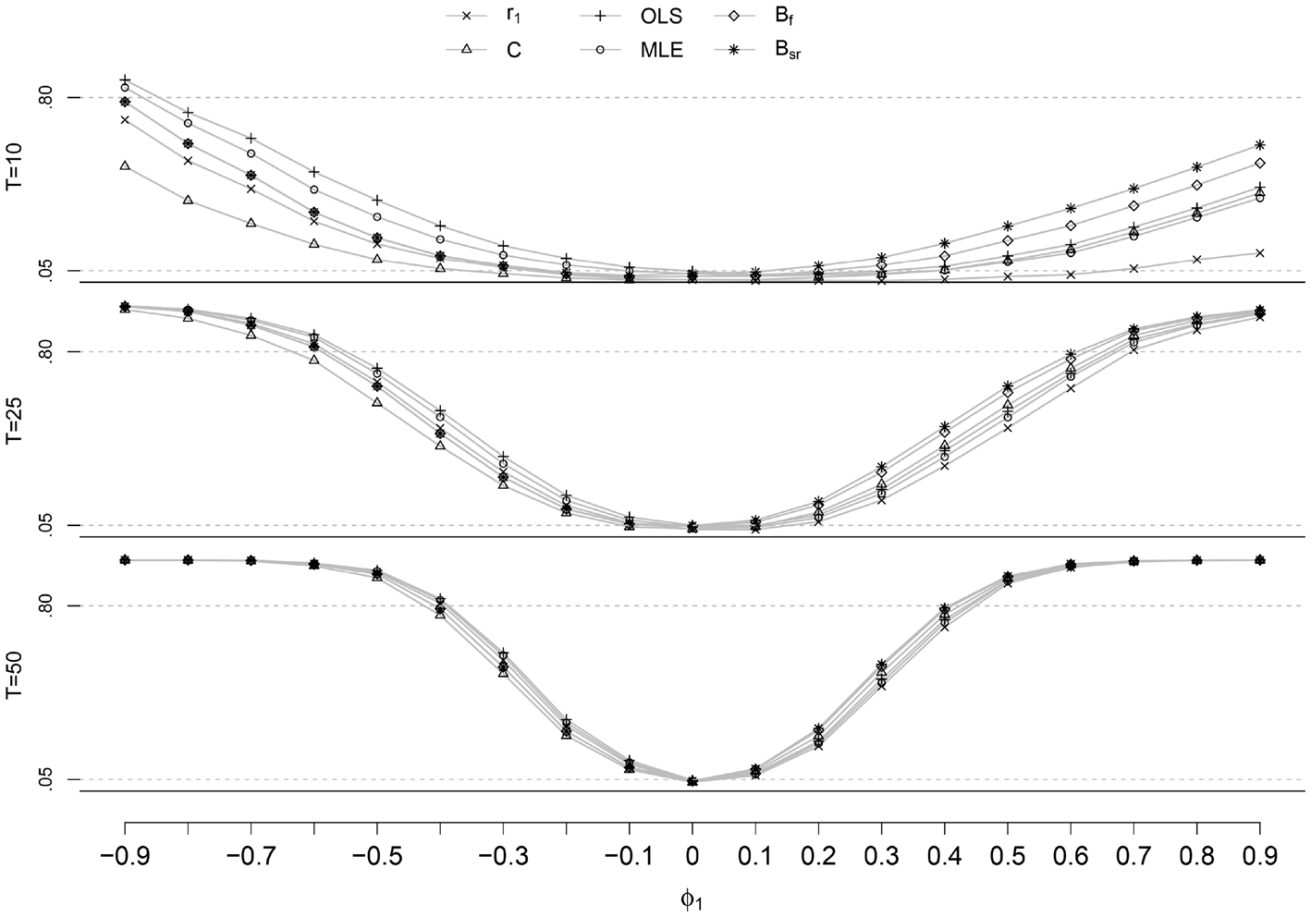
Power as a function of *ϕ* for the different estimation methods and *T* = 10, 25 and 50

### Rejection rate and power

The EPr of the different methods is presented in Fig. 3 for *T* = 10, 25 and 50, where the EPr at *ϕ* = 0 indicates the empirical rejection rate and the EPr at $\phi \ne 0$ the empirical power. As with the bias, the EPr for *T* = 40 and *T* = 100 are not shown due to their uninformative nature. When looking at the rejection rate, the empirical rejection rate approaches the nominal α as the length of the time series increases, as to be expected. The rejection rate for *T* = 10 is between 0.01 and 0.04 and for *T* ≥ 25 between 0.03 and 0.05, for most estimators. The only exception is the rejection rate for OLS-A at *T* = 40, which is 0.06. At *T* = 100, the MLE, B_sr_, OLS-S and B_f_ show a rejection rate of 0.050, which is equal to the nominal α of 0.05. For all practical purposes, the difference in rejection rates between estimation methods is negligable.

The power of the estimated *ϕ* shows a positive relation to the size of *T* and the absolute value of *ϕ*, as expected. When we would consider a minimal power of 0.80, for *T* = 10 this is only found for the estimators OLS and MLE, and at very low values of *ϕ*, i.e. $\phi \le -0.90$. For *T* = 25 and negative *ϕ*, the power is above 0.80 for $\phi \le -0.60$ for all estimators except for the C-statistic, which has a power above 0.80 for $\phi \le -0.70$; for positive values of *ϕ*, the B_sr_ shows a power above 0.80 for $\phi \ge 0.60$, for the other estimators this is for $\phi \ge 0.70$. For larger *T*, the power reaches 0.80 at lower values of *ϕ*; for *T* = 100, the power is 0.80 for $|\phi |\ge 0.30$.

The order of the estimation methods with regard to the power is consistent for the different values of *T*. The highest power for negative *ϕ* is shown by the OLS, for positive *ϕ* this is B_sr_. The lowest power for negative *ϕ* is shown by the C-statistic, for positive *ϕ* this is *ϕ*. In general, the difference in power between the methods becomes smaller as *T* becomes larger.

**Fig. 4:**
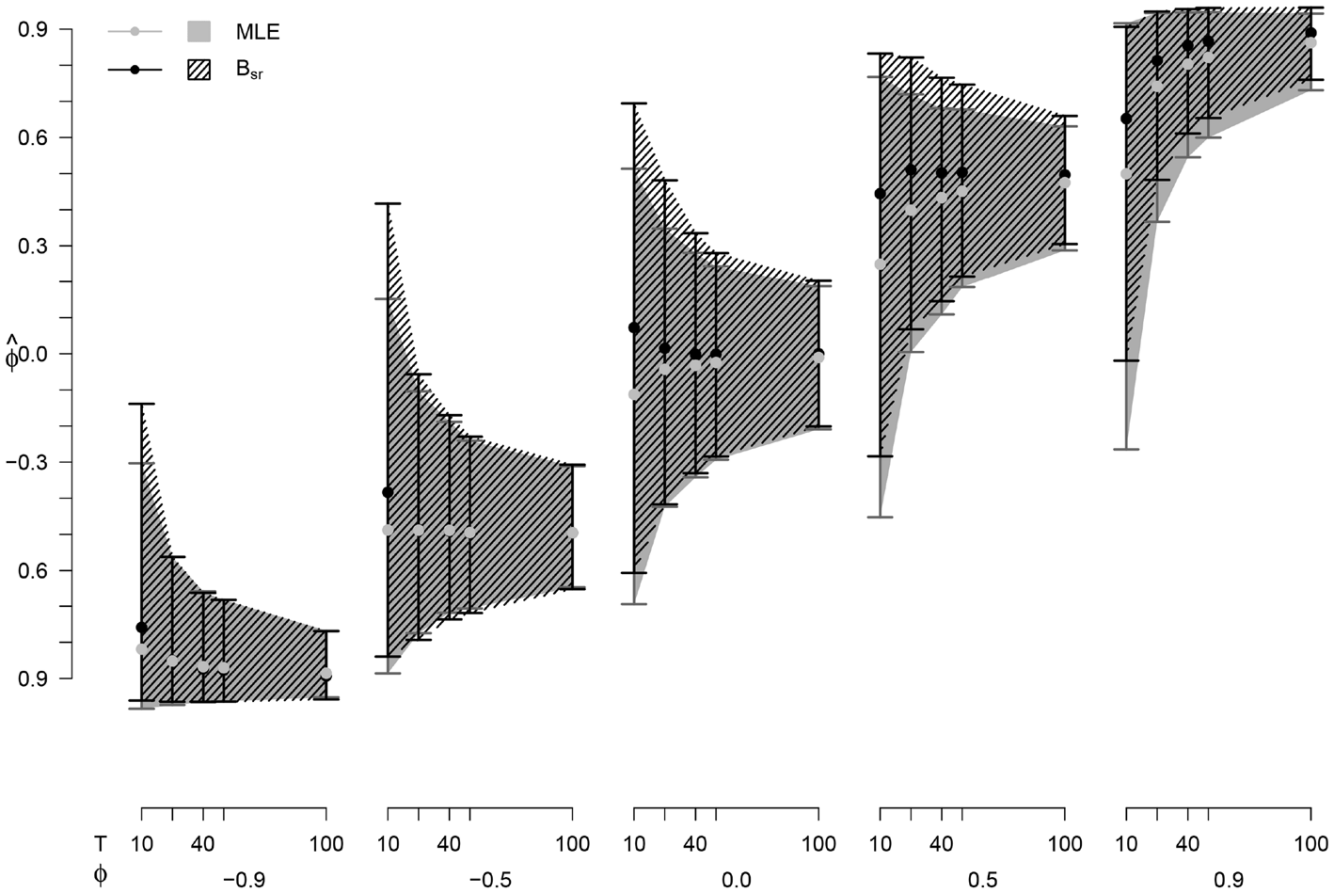
Mean of $\hat{\phi }$ (points) with a 95 % percentile interval (*lines*) for different values of *ϕ* and *T*

### Point and interval estimates for *ϕ*

The mean $\hat{\phi }$ with a 95 % estimation interval for the MLE and B_sr_ estimations can be seen in Fig. 4. We only present this for the MLE and B_sr_, since these methods show the lowest bias. As can be seen in Fig. 4, the 95 % intervals are larger for smaller values of *T*, indicating a larger variability in the $\hat{\phi }$, as would be expected. The strongest decrease in both variability and bias is from *T* = 40 to *T* = 25: for B_sr_ the bias decreases with up to 89 % and the variability with 29–51 %, for the MLE the bias decreases by 82 % and the variability by 34–51 %.

### Conclusion

In general, it may be concluded that the Bayesian B_sr_ and the frequentist MLE perform best in terms of bias, but not in terms of variability. With regard to empirical variability, the *r*_1_ performs best. For the bias of the estimated variability, the MLE performs best. Furthermore, the B_sr_ is favorable with regard to power for positive *ϕ*, showing only slight differences with the OLS estimator for a negative B_sr_. This leads us to continue with the MLE and the B_sr_ estimators for the misspecification study of this paper.

## Research design study 2: robustness

To study the robustness of the estimation methods, we misspecify the model. The data is still analysed as if they stem from an AR(1) model, but we generate the data using an ARMA(1,1) model. We generate data sets for two different sizes of *T*, namely 25 and 50. For *ϕ* and *θ*, we use parameters of −0.90 to 0.90 inclusive, taking steps of 0.15. Every condition consists of 5000 replications. Considering a fully crossed design, this yields 13 × 13 × 2 × 5000 = 1,690,000 datasets.

Again, across all conditions, *μ* is set to zero and $\sigma _e^2$ to one. We consider the same outcome measures for Study 2 as we did for Study 1.

## Results study 2

We successively present the results on the bias, empirical standard error, bias of the estimated standard error, rejection rate, power, and point and 95 % interval estimates. Note that when *θ* is zero, the simulated data follows an AR(1) model, rendering the results equal to the results discussed in the first study of this paper, apart from small deviations resulting from simulation variability.

As with the first study, we checked the $\hat{R}$ of the estimated parameters *ϕ*, *μ* and $\sigma _e$ of B_sr_. For each of the parameters, less than $0.14\,\%$ showed an $\hat{R}$ above 1.02, and less than $1.69\,\%$ showed an $\hat{R}$ above 1.01.

**Fig. 5:**
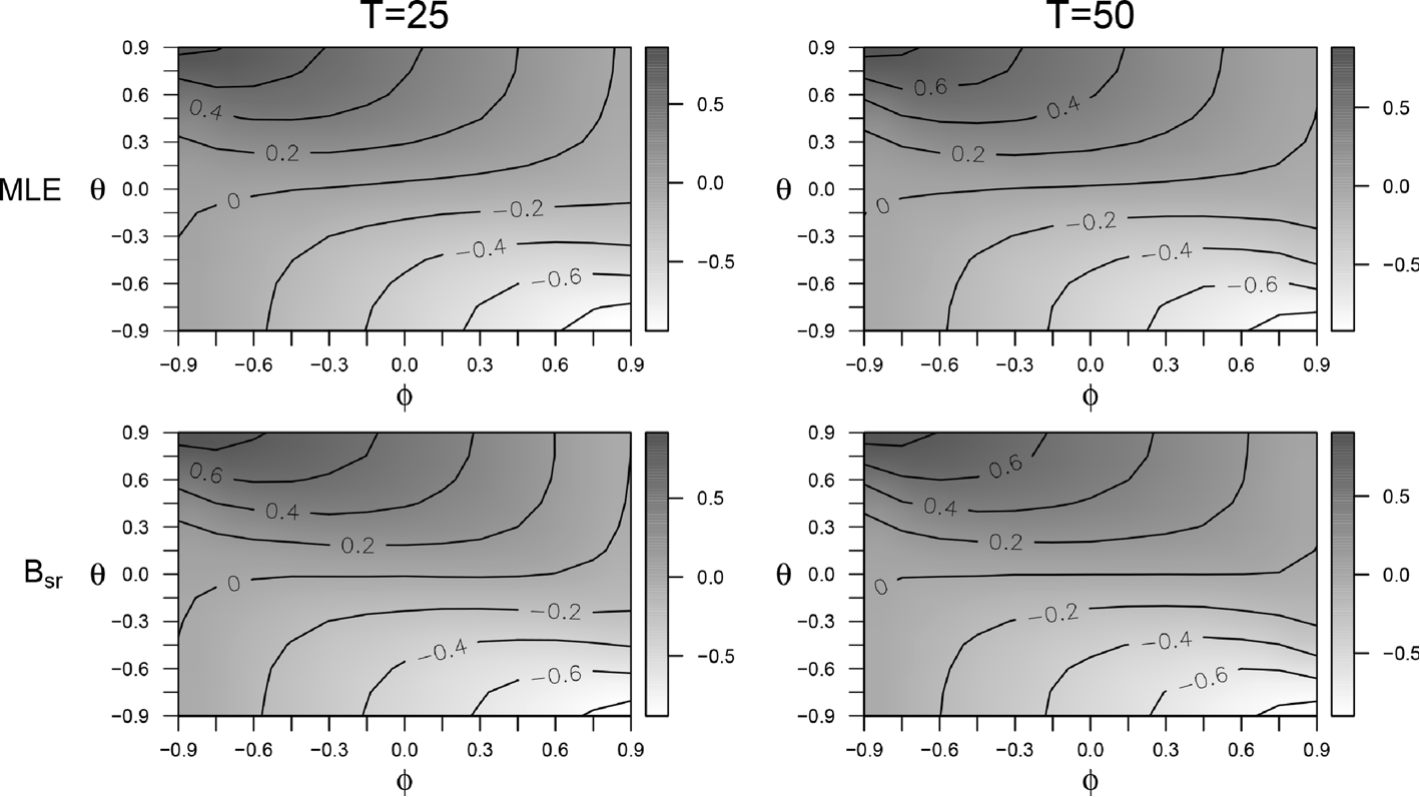
Heatmaps for the bias of $\hat{\phi }_{mle}$ for T = 25 and T = 50 (*top panes*) and the bias of $\hat{\phi }_{B_{sr}}$ for T = 25 and T = 50 (*bottom panes*)

### Bias

In Fig. 5, heatmaps for the bias of B_sr_ and MLE for *T* = 25 and *T* = 50 are presented, expressing the bias depending on the combination of *ϕ* and *θ*. The *θ* influences the bias in two ways: first, the bias is smaller when *θ* is close to zero, second, the bias gets larger when *θ* is further from *ϕ*.

The bias is also influenced by the value of *T* and the estimation method. When looking at *T*, in the MLE the bias for *T* = 50 is larger than the bias for *T* = 25, unless both *θ* and *ϕ* are negative. The difference between the bias of *T* = 50 and the bias of *T* = 25 ranges for MLE from −0.03 to 0.12 per condition. For the B_sr_, the bias for *T* = 50 is smaller than for *T* = 25, unless *ϕ* has a value above 0.30. The difference between the bias of *T* = 50 and *T* = 25 ranges for B_sr_ from −0.07 to 0.03 per condition. Comparing the estimation methods reveals that the bias is small, and in general slightly larger for the B_sr_ than for the MLE, with the largest difference being 0.11 for $\phi =0.60$, $\theta =0$, and *T* = 25. The difference between the estimation methods is larger for *T* = 25 than for *T* = 50.

**Fig. 6:**
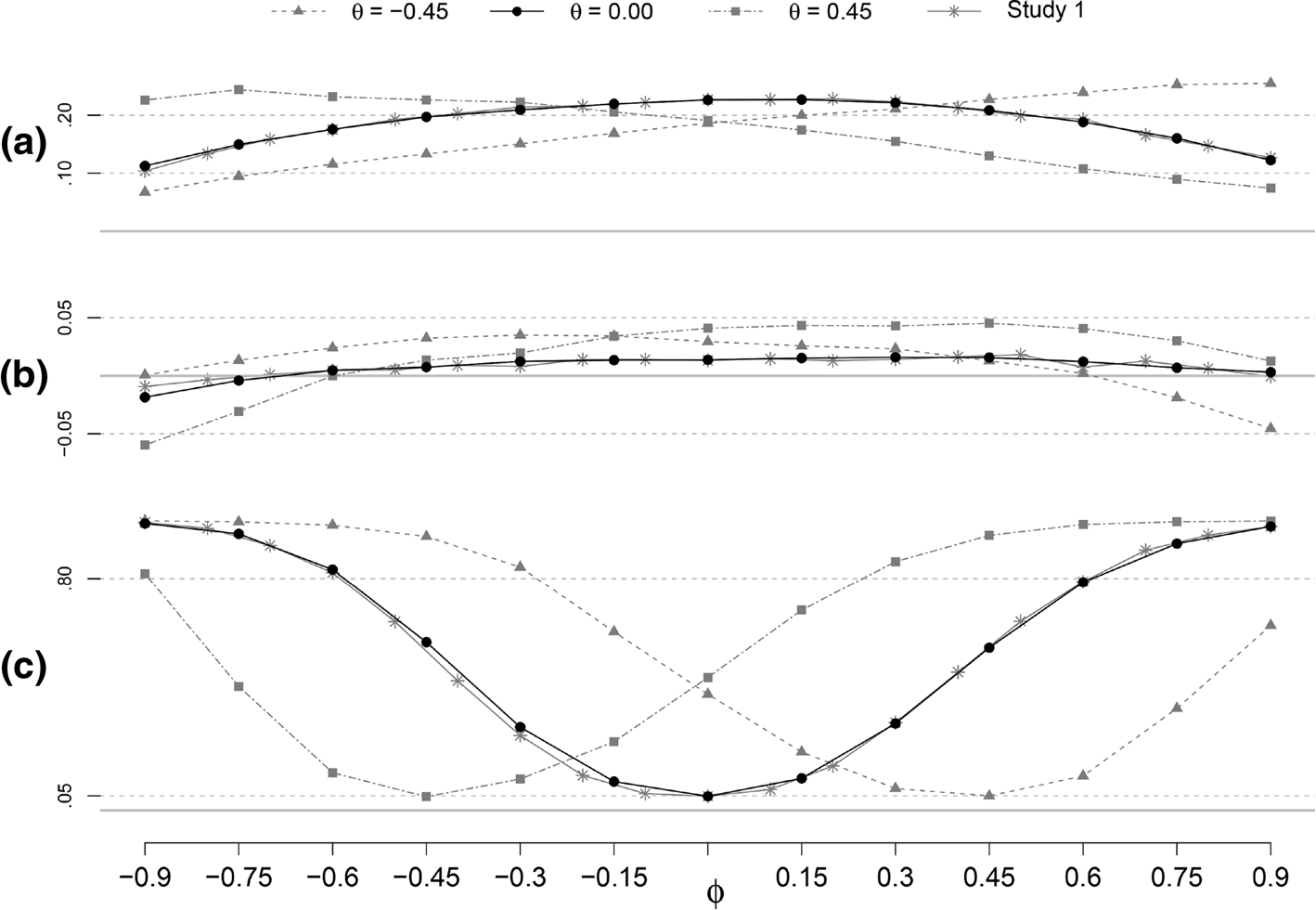
Empirical standard error (*panel a*), bias of the estimated standard error (*panel b*) and EPr (*panel c*) for B_sr_ with T = 25 as a function of *ϕ*

### Variability

Close inspection of the results for the variability and EPr for the B_sr_ and MLE estimators and the two lengths of *T*, revealed that the patterns are very similar across methods and different lengths of *T*. This prompted us to only present the results of B_sr_ and *T* = 25 in Fig. 6. However, we discuss any quantitative differences between the methods. For comparison purposes, we also plotted the $SD(\hat{\phi })$, the bias of $SE(\hat{\phi })$ and the EPr for B_sr_ and *T* = 25 of Study 1.

#### Empirical standard error: $SD(\hat{\phi })$

The empirical standard error ($SD(\hat{\phi })$), for B_sr_ with *T* = 25 and $\theta = -0.45, 0.00$, and 0.45, can be seen in Fig. 6 (panel a). Some differences between the $SD(\hat{\phi })$ over different values of *θ*, *ϕ* and *T* are found. First, the $SD(\hat{\phi })$ shows a positive slope over *ϕ* for negative values of *θ*, and a negative slope over *ϕ* for positive values of *θ*. Second, the $SD(\hat{\phi })$ is smaller for *T* = 50 compared to *T* = 25. For the MLE estimator the $SD(\hat{\phi })$ is up to 0.07 smaller for *T* = 50, for the B_sr_ the $SD(\hat{\phi })$ is up to 0.08 smaller for *T* = 50. When comparing methods of estimation, the $SD(\hat{\phi })$ of the B_sr_ is larger than the $SD(\hat{\phi })$ of the MLE, for both values of *T*. For *T* = 25, this differs up to 0.03, for *T* = 50 this differs up to 0.01 per condition.

#### Bias of the estimated standard error

The bias of $SE(\hat{\phi })$ for B_sr_ with *T* = 25 and $\theta = -0.45, 0.00$, and 0.45, can be seen in Fig. 6 (panel b). The bias of $SE(\hat{\phi })$ for most combinations of *ϕ* and *θ*, where $\theta \ne 0$, is positive and higher than the bias for the AR(1) data. Only for low values of $|\theta |$ combined with high values of $|\phi |$, the bias of $SE(\hat{\phi })$ is negative. The bias of $SE(\hat{\phi })$ is larger for *T* = 25 than for *T* = 50, for all methods and conditions, with differences up to 0.02 for both methods. Furthermore, the bias of $SE(\hat{\phi })$ is slightly larger for the B_sr_ estimation than for the MLE, with a maximum absolute difference of 0.01 for *T* = 25 and 0.03 for *T* = 50.

### Rejection rate and power

The EPr for B_sr_ with *T* = 25 and $\theta = -0.45, 0.00$, and 0.45, can be seen in Fig. 6 (panel c). When $\theta \ne 0$, the curve of the EPr shifts relative to the curve of the AR(1) data. For a negative *θ*, the curve shifts to the right, for a positive *θ*, the curve shifts to the left. In both cases, the amount the curves shifts is roughly equal to the absolute value of *θ*. This is the same for both methods, with the shape of the curve being dependent on *T*, as in Study 1. The differences between the methods are negligible.

**Fig. 7:**
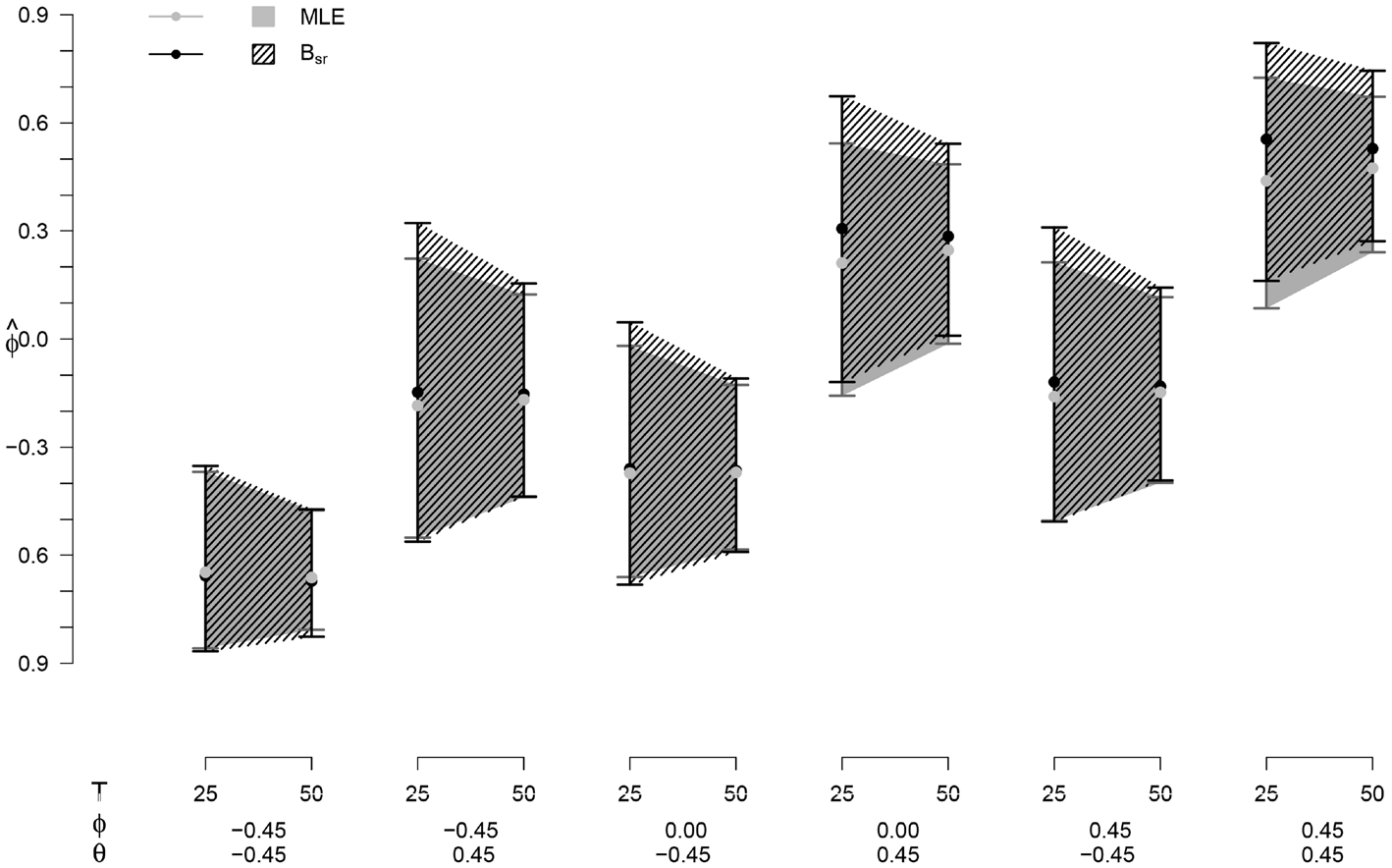
Mean of $\hat{\phi }$ (points) with a 95 % percentile interval (*lines*) for different values of *ϕ* and *T*

### Point and interval estimates for *ϕ*

The mean $\hat{\phi }$ with a 95 % estimation interval for the MLE and B_sr_ estimations are presented in Fig. 7. As can be seen in Fig. 7, the 95 % intervals are larger for smaller values of *T*, indicating a larger variability in the $\hat{\phi }$. For both methods, the decrease in the 95 % estimation interval is between 33 and 44 % from *T* = 25 to *T* = 50 for the different conditions. In most conditions, the 95 % estimation interval is larger and the mean $\hat{\phi }$ is slightly higher for the B_sr_ than for the MLE.

### Conclusion

We found that the further the *θ* deviates from zero, the larger the difference between the *ϕ* and $\hat{\phi }$ is. The B_sr_ shows a larger bias than the MLE when *θ* is further from zero, showing a larger influence of the *θ* parameter in the estimated *ϕ*. Furthermore, the observed variability is slightly smaller for the MLE, with the difference between B_sr_ and MLE being larger for *T* = 25 than for *T* = 50.

## Discussion

We compared five estimation methods for the autocorrelation: the *r*_1_, C-statistic, ordinary least squares, maximum likelihood estimation and Bayesian MCMC estimation. For the Bayesian MCMC estimation we used both a flat prior and a symmetrized reference prior, giving a total of six autocorrelation estimators. We compared these estimators with regard to bias, variability, rejection rate, power and point and 95 % estimation interval estimates. After this comparison, we selected the Bayesian estimation with symmetrized reference prior and the maximum likelihood estimator to use in a second study. In this small study we assessed the robustness of the methods against underspecification.

The results we found in the first study largely complied with the results from previous studies. For the bias for positive values of *ϕ*, we found the bias of the C-statistic and the OLS to be smaller than the bias of *r*_1_, as did previous studies (DeCarlo and Tryon 1993; Huitema and McKean 1991; Solanas et al. 2010). For the empirical standard error, we found smaller values for *r*_1_ than for OLS, as did Huitema and McKean (1991). The low rejection rate we found for the *r*_1_ and the C-statistic confirms to earlier studies (Huitema and McKean 1991; DeCarlo and Tryon 1993; Solanas et al. 2010). The power we found for the C-statistic is similar to the power found by Arnau and Bono (2001). However, the results of Solanas et al. (2010) with regard to power were only partly confirmed by our study: for negative *ϕ* we indeed found a higher power for OLS followed *r*_1_, than for the C-statistic. But for positive *ϕ*, we found a higher power for the C-statistic than for *r*_1_.

The first study showed a strong improvement in all measures for all methods between *T* = 10 and *T* = 25. This improvement continued, be it not as strong, for higher values of *T*. When comparing methods, B_sr_ showed the smallest bias. For the frequentist methods, this was MLE followed by the C-statistic. The smallest empirical standard error is shown by *r*_1_, the smallest bias of the estimated standard error is shown by the C-statistic, the OLS and the MLE. We further found that a small bias is often paired with a high empirical standard error. The power was rather low for all methods at the lengths of time series we considered. For *T* = 25, the power is below 80 % for all methods for *ϕ* between −0.5 and 0.5, for *T* = 100, the power is below 80 % for *ϕ* between −0.2 and 0.2. The differences between methods with regard to power are negligible. In research areas where effect sizes are small, this may pose a problem. Some studies use moving windows to assess the stability of parameter estimates over time. For these moving windows, these results indicate that a moving window of at least 50 time points is advisable, especially when the differences in parameters over time are small.

The first study was conducted to explore the differences between estimation methods for the autocorrelation in a single subject design. However, this is only a small step in a large research area. The next step may be to explore these results in multilevel or group analyses, thus when there is not one but multiple subjects per dataset. Another issue may be how the different methods respond to non-stationary data, i.e., $|\phi |> 1$.

In the second study, the robustness of the MLE and B_sr_ to underspecification was examined. In general, we confirmed the notion that the further the unmodelled parameter is from zero, the larger the influence of this parameter is on $\hat{\phi }$ (Tanaka and Maekawa 1984). As with the first study, the empirical standard error decreased when *T* became larger. However, the bias reacted differently for both methods: for the MLE, the bias became slightly smaller for most conditions, where the bias of B_sr_ became slightly larger for a larger *T*. The difference in performance for all measurements between the MLE and the B_sr_ was small for both values of *T*. It was shown that the bias, variability, rejection rate and power were all highly dependent on the value of the non-modeled parameter in the data, *θ*. This can be related to the fact that the autocorrelation of the error also has an influence on the autocorrelation of the total score.

The robustness study was rather small and specific, looking into only one possible way to misspecify the ARMA (1, 1) model. More options within misspecification should be explored to find how robust the estimation methods are with regard to under-, over- and misspecification. Important points are the influence of a misspecified error distribution or overspecification of the model.

In conclusion, we found that the best performing methods for autocorrelation estimation are the Bayesian estimator with symmetrized reference prior and the maximum likelihood estimator. The difference in performance between these two is, for all practical purposes, negligible. The results for the measurements improving greatly between *T* = 10 and *T* = 25 and continue to do so, but in a less spectacular fashion. For the misspecification study, we found the size of *θ*, the non-modelled parameter, to be vital for the performance of the estimation methods. The differences between lengths of the series and estimation methods was of lesser influence on the results.
